# Retaining the general practitioner workforce in England: what matters to GPs? A cross-sectional study

**DOI:** 10.1186/s12875-015-0363-1

**Published:** 2015-10-16

**Authors:** Jeremy Dale, Rachel Potter, Katherine Owen, Nicholas Parsons, Alba Realpe, Jonathan Leach

**Affiliations:** Warwick Medical School, Coventry, CV4 7AL UK; Davenal House Surgery, Bromsgrove, Worcestershire B61 0DD UK

## Abstract

**Background:**

The general practice (GP) workforce in England is in crisis, reflected in increasing rates of early retirement and intentions to reduce hours of working. This study aimed to investigate underlying factors and how these might be mitigated.

**Methods:**

GPs in central England were invited to participate in an on-line survey exploring career plans and views and experiences of work-related pressures. Quantitative data were analysed using logistic regression analysis and principal components analysis. Qualitative data were analysed using a thematic framework approach.

**Results:**

Of 1,192 GPs who participated, 978 (82.0 %) stated that they intend to leave general practice, take a career break and/or reduce clinical hours of work within the next five years. This included 488 (41.9 %) who intend to leave practice, and almost a quarter (279; 23.2 %) intending to take a career break. Only 67 (5.6 %) planned to increase their hours of clinical work.

For participants planning to leave practice, the issues that most influenced intentions were volume and intensity of workload, time spent on “unimportant tasks”, introduction of seven-day working and lack of job satisfaction.

Four hundred fifty five participants responded to open questions (39128 words in total). The main themes were the cumulative impact of work-related pressures, the changing and growing nature of the workload, and the consequent stress.

Reducing workload intensity, workload volume, administrative activities, with increased time for patient care, no out-of-hour commitments, more flexible working conditions and greater clinical autonomy were identified as the most important requirements to address the workforce crisis. In addition, incentive payments, increased pay and protected time for education and training were also rated as important.

**Conclusions:**

New models of professionalism and organisational arrangements may be needed to address the issues described here. Without urgent action, the GP workforce crisis in England seems set to worsen.

**Electronic supplementary material:**

The online version of this article (doi:10.1186/s12875-015-0363-1) contains supplementary material, which is available to authorized users.

## Background

The General Practice (GP) workforce in the UK has been declared to be at “crisis point” [1]. There are difficulties in both recruiting doctors to GP specialist training [2] and an increasing trend to part-time working and earlier retirement [3, 4]. As with past GP workforce crises [5, 6], declining job satisfaction is a major contributing factor [7–9]. The recent British Medical Association (BMA) national survey of GPs reported 34 % GPs intending to retire within the next 5 years and 17 % hoping to change to part-time work, with workload intensity and volume being the predominant factors driving these intentions; 16 % said that they thought that their stress was significant and unmanageable.[10].

The recent policy document “Building the workforce - the New Deal for GPs” [11] proposes measures to address the current crisis, such as mentoring schemes and portfolio careers in addition to exploring workload interventions. However, the relevance of such measures to the issues that are driving GPs to contemplate leaving clinical practice needs further investigation. Furthermore, the British Government’s commitment to 7-day access to GPs by 2020 [12] risks exacerbating the workforce issues.

In this study, we used quantitative and qualitative methods to investigate factors that are affecting GPs’ work experience and influencing their plans to leave, reduce hours and/or take a career break in the near future. In addition, we sought to identify changes that might encourage GPs to remain active in the workforce. We surveyed all GPs working within a region, so enabling investigation of how work-related issues affect GPs at different stages of the career path and the measures that may be needed to address this.

## Methods

### Setting

The West Midlands region of England. This region is geographically, economically and ethnically diverse, from the urban central areas of the West Midlands conurbation (which includes the cities of Birmingham, Coventry and Wolverhampton) to the rural counties of Shropshire, Worcestershire and Herefordshire. In the 2011 national census, its population was 5.6 million. In 2013, there were 3667 GPs working in the West Midlands of whom 28.4 % were aged 50–59 years and 12.2 % over 60 years [13].

### Questionnaire

We designed a questionnaire for completion online, drawing on questions used in previous GP workforce surveys [3, 4]. Questions covered respondent demographics, practice characteristics, work-related morale and job satisfaction, career intentions and, for participants who reported that they intended to leave general practice or take a career break within the next 5 years, factors that had influenced their decision or might reverse this intention. The latter were drawn from issues that have been identified as potentially influencing retention in general practice in recent media coverage and policy documents [14]. The importance of each of these factors was rated using ordinal scales from 1 (‘not important’) to 5 (‘very important’). Participants had the option to add free text comments, and we invited all respondents to add any further comments at the end of the questionnaire.

The survey was piloted with a group of GPs to ensure clarity and that it was simple to navigate and complete.

### Population

We aimed for all GPs in the West Midlands to participate in the study. However, due to data protection and governance regulations, it proved impossible to obtain a complete list of contact information for GPs. Instead, we asked the relevant National Health Service (NHS) bodies who commission general practice services to email all practice managers asking them to circulate details of the study to GPs in the practice and encourage them to participate in the survey. In addition, we asked the Royal College of GPs Midland Faculty to email its members, and Health Education West Midlands to email GP trainers and course organisers. We requested that reminder emails be distributed after 2 weeks. The questionnaire was available for completion on-line between 8/12/2014 and 16/01/2015.

### Analysis plan

#### Quantitative data

Means and standard deviations of questionnaire responses were used to characterise the survey population and compare to regional norms. Logistic regression analysis was used to quantify associations between both the intention to remain in general practice beyond 5 years and take a career break and GP characteristics (age, gender, place of qualification, employment status, past and current portfolio roles in addition to main employment, length of time in practice, working hours) and practice characteristics (size, number of GPs and location). A stepwise search algorithm, using both forward and backwards selection, was used to identify statistically significant factors using the Akaike Information Criterion (AIC) as a measure of the relative quality of a statistical models under test.

To understand the intensity with which factors contribute to the intention to leave practice or take a career break, and how they are associated with practice and individual characteristics, we grouped items into “workplace influences” and “individual motivators”. Workplace influences comprised of items that are directly associated with work-related responsibilities and experience: volume and intensity of workload, clinical autonomy, administrative burden, skill mix, flexibility of hours, out-of-hours responsibilities, time for patient contact and revalidation. Individual motivators comprised of items that are associated with personal attributes, aspirations and rewards: age, family commitments, ill health, financial incentives, changes to pension taxation, protected time for education and training, annual leave, career breaks and sabbaticals.

We then used principal components analyses (PCA) to understand the importance of factors contributing to these determinants, extracted the first two principal components (PCs) and then used multiple linear regression to model each PC using GP and practice explanatory variables in an analogous manner to that used for the logistic regression model (see above).

Tests were considered to provide evidence for a significant difference if *P* < 0.05 (5 % significance level) and all statistical analysis was implemented in R [15].

#### Qualitative data

We included a number of open questions in the survey to allow respondents to clarify their responses and to identify any issues not covered by the closed questions. The free text comments were managed using NVivo 10 and we used a thematic framework approach to analyse the data [16]. Two authors (RP and AR) separately coded a sub-set of comments to explore emergent themes and together devised a coding framework to describe the thematic content of the comments. The coding was applied until it was judged that no new information was acquired. The higher order categories were linked to the results of the survey analysis and were used to supplement and expand the interpretation of the quantitative data.

#### Ethical approval

NHS ethical approval was not required as the research did not involve patient participants (http://hra-decisiontools.org.uk/ethics). Ethical approval was provided by the University of Warwick’s Biomedical Sciences Research Ethics Committee.

## Results

In total, 1192 GPs participated. The gender distribution was representative of the West Midlands GP population (55.7 % male, 44.3 % female) [13], the age distribution was broadly representative, except for over-representation of participants aged between 50–59 years (38 % respondents compared to 28 % regionally) and those trained in UK/Ireland (85.8 % respondents; 72.6 % regionally), and under-representation of participants from practices with one or two GPs (8.4 % respondents; 36.5 % regionally). Most participants were GP principals (74.9 % compared with 72.4 % regionally). Almost half (49.9 %) worked 41 h or more in a typical week. Almost two-thirds had at least one externally accountable role (e.g. appraiser, trainer, undergraduate tutor, specialist clinical role) outside their clinical and managerial responsibilities within the practice (Additional file 1: Table S1).

Not all respondents answered all questions; the percentages given below refer to the number who answered each question. The logistic regression analysis and PCA were based on complete case data. For the logistic regression this was 86 % (1027/1192) of the full study population and for the PCAs ranged between 76 % (371/488) and 80 % (391/488) of GPs who intend to leave general practice in the next five years.

Additional free text comments were provided by 455 (38.2 %) participants in response to one or more questions about factors contributing to their decision to leave or potentially remain in practice. There were 39128 words in total, with individual comments ranging from 4 to 554 words (mean 86 words).

### Intention to leave practice, retire or take a career break

Table 1 shows GPs’ career intentions for the next 5 years. In all, 978 (82.0 %) participants intend to leave general practice, take a career break and/or reduce their clinical hours of work within the next five years. This included 488 (41.9 %) who intend to leave practice, and almost a quarter (279; 23.2 %) who stated that they intend to take a career break.

**Table 1:** Participants’ intentions to remain in GP Workforce (% relates to number answering each question)

Intentions	Frequency (percent)
Work plans for the next five years	537 (45.1 %)
Reduce hours of clinical work	67 (5.6 %)
Increase hours of clinical work	216 (18.1 %)
Reduce management responsibilities	201 (16.9 %)
Increase management responsibilities	150 (12.6 %)
Reduce teaching/training/research responsibilities	206 (17.3 %)
Increase teaching/training/research responsibilities	161 (13.5 %)
No plans to change	113 (9.5 %)
Intention to remain in general practice beyond the next five years	
Yes	676 (58.1 %)
No	488 (41.9 %)
Intention to work after retirement	
Full-time	11 (3.0 %)
Part-time	102 (27.6 %)
Will not work after retirement	152 (41.1 %)
Unsure	105 (28.4 %)
Intention to take a career break from general practice within next 5 years	
Yes	267 (23.2 %)
No	883 (76.8 %)
Reason for taking a career break	
Starting a family/young children	46 (17.2 %)
Looking after older children	12 (4.5 %)
Caring for someone	7 (2.6 %)
Travel/work abroad	125 (46.8 %)
Study or research	20 (7.5 %)
Other	57 (21.3 %)
Length of planned career break	
Less one year	90 (36.1 %)
Between 1–2 years	50 (20.1 %)
More than 2 years	27 (10.8 %)
Unsure	82 (32.9 %)

Of those planning to leave practice within 5 years, 65.6 % were male, 64.3 % aged between 50–59 years and 84 % are GP principals. Of those planning to retire, 30.6 % planned to work in general practice after retirement, and a further 28.4 % were unsure about whether they would continue to work.

Stepwise logistic regression analysis identified age, gender, length of service and lack of additional portfolio roles as significant predictors of a GP’s intention to leave general practice within five years (Additional file 1: Table S2a). Unsurprisingly, GPs aged 50 years or over are more likely, estimated odds ratio 9.2 (95 % CI 6.0 to 14.3; *p* < 0.001), as are those with 10 years or more in practice (2.1, 95 % CI 1.3 to 3.5; *p* = 0.003). For GPs under 50 years of age, male GPs are more likely to express an intention to leave practice than female GPs; estimated odds ratio 0.37 (95 % CI 0.22 to 0.61; *p* < 0.001). Interestingly, GPs with a portfolio career (one or more accountable roles in addition to their main practice responsibilities) are less likely to express an intention to leave practice (0.56, 95 % CI 0.39 to 0.80; *p* = 0.001).

Logistic regression analysis identified age and number of hours worked as statistically significant predictors of a GP’s intention to take a career break within five years (Additional file 1: Table S2b). GPs aged 50 years or over were less likely to think about taking a career break, estimated odds ratio 0.62 (95 % CI 0.46 to 0.83; *p* = 0.001), than those aged under 50 years. GPs who worked more than 40 h a week were more likely to think about a career break, estimated odds ratio 1.50 (95 % CI 1.12 to 2.01; *p* = 0.006), than those who worked fewer hours.

### Factors influencing intention to leave general practice

For participants who stated an intention to leave general practice within the next five years (*n* = 488), the items (rated from 1 ‘not important’ to 5 ‘very important’) that had the greatest influence were *intensity of workload* (mean = 4.6; sd =0.86), *volume of workload* (4.5; 0.92), *time spent on unimportant tasks* (4.4; 0.97), *introduction of a seven-day working week* (4.2; 1.2) and *job satisfaction* (4.2; 1.1). Individual motivators that emerged as important with a mean score of greater than 3 comprised *changes to pension taxes* (3.3; 1.5) and *age* (3.2; 1.4) (Table 2).

**Table 2:** Factors i) influencing intentions to leave clinical practice and ii) that might lead to retention in practice

Factors influencing decision to leave general practice			Factors that might retain GPs in practice		
(1 = not important to 5 = very important)			(1 = not important to 5 = very important)		
*Workplace influences*					
	mean	sd		mean	sd
Volume of workload	4.5	0.92	Reduced intensity of workload	4.5	0.91
Intensity of workload	4.6	0.86	Reduced volume of workload	4.4	0.91
Too much time spent on unimportant tasks	4.4	0.97	Less administration	4.3	1.0
Introduction of 7 day a week working	4.2	1.2	Longer appointment times	4.2	1.1
Reduced job satisfaction	4.2	1.1	No out of hours commitments	3.9	1.5
Lack of time for patient contact	4.1	1.0	More flexible working conditions	3.5	1.4
Poor flexibility of hours	3.0	1.3	Greater clinical autonomy	3.3	1.4
Revalidation	2.6	1.4	Improved skill-mix in the practice	3.0	1.9
			Shorter practice opening times	2.9	1.4
			Option to work term time only	1.8	1.3
*Individual motivators*					
Changes to pension taxation	3.3	1.5	Incentive payment	3.7	1.4
Age	3.2	1.4	Protected time for education and training	3.6	1.3
Family commitments	2.6	1.0	Increased pay	3.1	1.5
Ill health	1.6	1.0	Additional annual leave	2.9	1.4
Embarking on career outside general practice	1.8	1.3	Opportunity for a sabbatical	2.7	1.5
Planned career break	1.3	0.89	Extended interests e.g. CCG role	2.3	1.4
			Introduction of ‘Twenty Plus’	2.3	1.4
			Reintroduction of the flexible careers scheme	2.0	1.3
			Expansion of GP retainer scheme	1.9	1.3

Two principal components (PCs) were identified which together account for 52 % of the variance in the workplace-related influences (Additional file 1: Table S3a). The first PC was *overall workload*, a weighted combination of all work-related factors. The second PC was *working conditions*, the importance given to flexibility and job satisfaction relative to volume and intensity of work and the other items.

A linear regression model indicated that *overall workload* is more important to GPs aged under 50 years than those aged 50 years or over, and more important to GP principals than non-principals (*p* < 0.001 and *p* < 0.001 from likelihood ratio tests (LRT) of nested models).

W*orking conditions* become relatively less important with increasing age, are more important to males than for females, and more important to those who are a GP appraiser (*p* < 0.001, *p* < 0.001 and *p* < 0.001 from LRT). In addition, the size of practice (number of GPs) is inversely associated with the importance of *working conditions* (*p* < 0.001; LRT).

There were two principal components that accounted for 61 % of the variance in non-workplace related scores (Additional file 1: Table S3b). These PCs related to w*ork*-*life flexibility* and *personal development*. W*ork*-*life flexibility* is a weighted mean of all non-work related factors and *personal development* represents the difference in importance of scores between those factors related to training and education opportunities and other non-work related factors.

*Work*-*life flexibility* is less important with increasing age, more important for female GPs, less important for increased length of time in practice, less important for GPs working in large practices than small practices, and more important for GPs with portfolio careers (*p* = 0.002, *p* = 0.002, *p* = 0.001, *p* = 0.006 and *p* = 0.020; LRT).

P*ersonal development* is more important for female GPs, GPs with over 10 years’ service, and for GP principals than non-principal GPs (*p* = 0.013, p = 0.012 and *p* = 0.029; LRT from linear regression analysis).

### Features of work-related pressure

Free-text comments detailed the key aspects of general practice workload that participants perceived as contributing to excessive work-related pressure. These are summarised below and presented with example quotes in order of the frequency with which they were described.

#### Growth in patient expectations and demand

Patients’ expectations are increasing in ways that outstrip what can be provided within NHS general practice. This was felt to reflect an increasingly consumerist approach to healthcare.

“*Patient expectations have increased. Some of which reflects change in society which we all must adapt to, but the last few years there had been a sea change in expectations*” (ID 97, age 30–39, male, principal).

*“An increasing demand on the NHS by the public who are not educated in terms of seeking appropriate help, and who do not attempt self-care, but are all too willing to take a consumerist approach to health care and complain and criticise the profession at every available opportunity”* (ID 86, age 30–39, female, principal).

#### Recruitment and retention difficulties

This included the difficulties experienced in filling vacant GP posts and attracting trainees into general practice, the expense of employing practice staff and locums and the decreasing number of young GPs aspiring to partnership. The gaps in the workforce were compounded by GPs leaving practice due to early retirement, reducing hours, taking career breaks or leaving the profession.

*“I worry about the future of General Practice with a workforce crisis due to experienced GP’s leaving the profession or retiring early and newly qualified GP's choosing to work abroad or only wanting to work part time”* (ID 130, age 40–49, female, principal).

*“I know a few who either left partnership to become salaried or left the UK altogether. Our practice had more GPs leaving than staying and advertising jobs only resulted in one applicant on several occasions”* (ID 414, age 30–39, female, practice-employed salaried).

#### Burgeoning administration and bureaucracy

Participants described an “exponential” increase in administration and bureaucracy, consuming hours of time with limited perceived benefit to patients, leading to work regularly being undertaken outside normal working hours.

*“More and more now though I feel as if getting the information onto the computer is more important to listening to the patient”* (ID 210, age 40–49, female, principal).

*“I just want to get on with caring for the patients, I am fed up with the endless pointless paper exercises that we are forced to do to maintain our income”* (ID 375, age 40–49, female, principal).

*“My energy runs out at about 9 h of continuous use of my prefrontal cortex and I am finding the 12 h days needed to deal with all my inboxes and the marathon Sunday sessions to complete my admin are rather draining”* (ID 257, age 50–59, male, principal).

#### Growth in additional roles, responsibilities and meetings

Extra responsibilities within and outside practice (such as CCG-related responsibilities) resulting in increased number of meetings to attend, less time for patient care or to pursue interests such as teaching, research or training.

*“I still love the day to day clinical work but there is less time to spend with patients due to the increasing number of meetings I need to attend - safeguarding network meetings quarterly, safeguarding practice meetings with HV quarterly, GSF palliative care network meetings quarterly, GSF practice meetings 4–6 weekly, prescribing meetings, preparing for CQC, writing policies etc., etc*.” (ID 371, age 50–59, female, principal).

#### Transfer of work from secondary care

There has been a substantial transfer of unfunded work from secondary care, without commensurate funding or resources.

*“The amount of tasks passed back from hospital without funding is increasing alarmingly, particularly as patients are discharged so very early from operations or episodes of illness*” (ID 223, age 40–49, male, principal).

*“The administrative workload has increased to unmanageable levels over the past 3–4 years. Transfer of unfunded work from secondary care is escalating… All of the full time partners are desperate to reduce their commitment…”* (ID 366, age 50–59, male, principal).

*“Currently still very much enjoying the clinical contact with patients but very dispirited due to overwhelming workload as work is passed out to Primary Care from Secondary Care with no funding to employ staff to do it. Obviously patients can no longer get normal GP appointments as a large number of our appointments are now used up doing hospital outpatient duties”* (ID 391, age 50–59, male, principal).

#### Increasing complexity and chronic ill health

Patient care is becoming increasingly complex, reflecting factors such as the aging population, increasing chronic health needs, mental health, multi-ethnicity etc. This was felt to be leading to unmanageable pressure within the current model of 10-min consultations.

*“The number of very complex patients and conditions we now have to manage in 10 min consultations is impossible”* (ID 200, age 40–49, male, principal).

#### Revalidation and regulatory assessment

Revalidation, CQC and other assessments were felt to involve lengthy preparatory time and considered to be largely tick-box exercises, providing little of benefit in their current format.

*“I must be one of the few GPs who support this [GP appraisal] in principle, I understand why it is necessary. But does the process have to be such an incredible meal! The time required to assemble the evidence is absurd and highly demotivating”* (ID 153, age 50–59, female, principal).

*“The process was stressful and devoid of much real meaning. Tick box, limited scope for professionalism, over emphasis on legalism and managerialism. The whole approach was all about defeating computer systems”* (ID 306, age 70+, male, freelance GP).

#### The introduction of seven-day working in general practice

Several comments highlighted that the government’s intention to introduce seven-day working in general practice was likely to exacerbate the workforce crisis.

*“We are being told that we will be seeing patients from 8 to 8, 7 days a week, but we do not have enough GPs to cover the normal working schedule!”* (ID 670, age 30–39, female, principal)

*“I am utterly demoralised with the current situation with general practice. I can only see the job worsening - particularly with 7 day working”* (ID 56, age 30–39, male, practice-employed salaried)

*“Increasing patient access seems to be the focus of politicians (not sure whose going to be around to see these patients though), and I'm still unclear why GP needs to available in this way- it is not an emergency service. Let's face it, doctors do not choose GP so they'd be forever at work- one of the attractions was the lack of weekends and extended weekday hours”* (ID 475, age 30–39, female, role not disclosed)

### Emotional impact of working as a GP

A further issue that emerged, but was not directly addressed by the survey’s questions, was the *emotional impact* associated with work-related pressures. Participants described feeling stressed, exhausted, disillusioned, frustrated, overwhelmed and burnt out, despite still enjoying many aspects of patient care, as reflected below:

*“Despite loving the fabric of my job, my patients, and celebrating a superb team of staff around me I have never felt demoralised as I do currently. If there were a realistic financial option of retiring in the next 5 years I would take it, not because I want to stop working but because my current conditions are destroying my health and psychological wellbeing…”* (ID 195, age 50–59, male, role not disclosed).

*“…I have recently negotiated with my practice to reduce my hours from 3 to 2 days per week. While I know it sounds pathetic I really don't feel I can cope working 3 days anymore. The pressure is tremendous. I get home after a 10 h day and feel as if I have been hit round the head with a brick…I am only 40 yet feel burnt out. It is a miserable situation”* (ID 375, age 40–49, female, practice-employed salaried GP).

*“There are several Drs I know that are burning out, including a colleague who is on long-term sick leave now for this. This is a wonderful experienced GP who we are now likely to lose forever because they feel they cannot take the pressure anymore” (*ID 146, age 40–49, male, principal).

A key concern that many participants felt very strongly about was the effect of the ‘constant’ and ‘relentless’ negative portrayal of GPs by government and media which was damaging their morale and professional identity:

*“I find the negative image perpetually portrayed by the media and government extremely demotivating fuelling patient expectation and demand which is simply unachievable”* (ID 418, age 50–59, male, principal).

*“Having worked as a GP for many years I feel this speciality is heading for a crisis. Morale is extremely low as GPs are battered by the media and blamed for the increased demands on OOH care, despite the fact that I feel we are working harder than ever”* (ID 62, age 30–39, female, principal).

*“After 33+ years in GP I've never known morale to be so low. We are constantly being undermined by the government, bad press CQC etc. and the CQC stuff is the final straw… Nobody has stood up for GPs to say that it is too much, so I am retiring earlier than I had planned. Perhaps when there are no GPs left someone will ask why?”* (ID 153, age 60–69, male, principal).

### Cumulative impact of work-related pressures on GPs

The combined and cumulative effect of the work-related pressure, emotional impact and low morale was emphasised by many participants.

*“I cannot see myself continuing to work the hours I am currently with the increasing pressures, spiralling workload and disparity between the patient demand and capacity we can realistically can provide…I am considering leaving my partnership and working flexible sessions…The idea of this being possible until my late 60s when my NHS pension is earned is ridiculous”* (ID 347, age 40–49, female, principal).

*“I have had enough and am in the process of moving permanently abroad. I am 35 and been a partner for over 4 years. What a waste… Bullying by central government, micromanagement, attack dog GMC that clinicians are "afraid of", UK power shift that we are second class citizens to the public at large, increasing workload and risk for decreasing returns. I could weather most of this but for the lack of respect the powers that be and the public have for us. We have been de-professionalised, de-humanised and de-moralised”* (ID 241, age 30–39, male, principal).

*“I've had a wonderful 29 years as a GP…. I leave in 6 weeks aged 56….Any fool can see that the responsibility of the job is unlimited and the people trying to do it are highly committed and so vulnerable. The damage done to morale by this government may well prove irreparable…. I work 8 am-7 pm nonstop every day, come in at weekends, work out of hours and have been doing this for nearly 3 years, I need support not relentless criticism”* (ID 47, age 50–59, male, principal).

*“I am part time in a large well organised practice with a great skill mix, good pay and plenty of annual leave. It is the constant negative press and unrealistic expectations of politicians and public, constant reorganisation and threat of further rule changes that wear GPs down. I’ve had (nearly) enough!”* (ID 198, age 50–59, male, principal).

### Improving working conditions to retain the GP workforce

From the quantitative measures, the key items that might reverse the intention to leave practice were: *reduced workload intensity* (mean = 4.5; sd = 0.91), *reduced workload volume* (4.4; 0.91), *less administration* (4.3; 1.0), *greater time spent with patients* (4.2; 1.1), *no out-of-hour commitments* (3.9; 1.5), *more flexible working conditions* (3.5; 1.4) and *greater clinical autonomy* (3.3; 1.4). Individual motivators that were also rated as important (mean rating of greater than 3) comprised *incentive payments* (3.7; 1.4), *increased pay* (3.1; 1.5), *protected time for education and training* (3.6; 1.3). *Additional annual leave* (2.9; 1.4) and *opportunity for a sabbatical* (2.7; 1.5) had mean scores of just below the mid-point.

Initiatives that are being considered as part of the “Building the workforce - the New Deal for GPs” policy to retain GPs in practice and encourage returners were viewed as having less importance: *introduction of ‘Twenty Plus’ scheme* (similar to Royal College of GPs ‘First Five’ programme [14]) (2.3; 1.4), *reintroduction flexible careers scheme* (2.0; 1.3) and *expansion of retainer scheme* (1.9; 1.3) (Table 2).

#### Workplace influences

Two principal components *overall workload* and *working conditions* account for 59 % of the variance in work-related scores (see Additional file 1: Table S4a.). *Overall workload* is more important for GPs aged < 50 years (*p* = 0.023), and likewise *working conditions* is relatively less important with increasing age as an encouragement to remain in practice (*p* = 0.002; LRT).

#### Individual motivators

Two principal components w*ork flexibility* and *personal development* account for 61 % of the variance (see Additional file 1: Table S4b). W*ork flexibility* is a weighted mean of all non-work related factors and *personal development* represents the difference in importance of scores between those factors related to training and education opportunities and other non-work related factors.

*Work flexibility* is less important with increasing age, and more important for female GPs, less important for increased length of time in practice, less important for GPs working in large practices than small practices, and more important for GPs with portfolio careers (*p* = 0.002, *p* = 0.002, *p* = 0.001, *p* = 0.006 and *p* = 0.020; LRT). P*ersonal development* is more important for female GPs, GPs with ≥ 10 years’ service, and for GP principals than non-principal GPs (*p* = 0.013, *p* = 0.012 and *p* = 0.029; LRT from linear regression analysis). Increased pay is less important for GPs aged ≥ 50 years than those aged < 50, and less important for GPs at larger than smaller practices (*p* < 0.001 and *p* < 0.001; LRT).

Participants’ comments emphasised the importance of the NHS, government and representative professional bodies adopting a more ‘realistic’ approach in order to reduce workload, administrative activity and patients’ expectations, and increasing the funding available to general practice.

*“…change in attitude from politicians and press blaming all the NHS problems at the door of general practice. No more unrealisable promises to the public!”* (ID 446, age 50–59, male, role not disclosed).

*“I would like political interventions and policies to be based on scientific evidence, not whim - like NHS Health Checks or the Cancer Fund”* (ID 354, age 50–59, female, principal).

*“A more realistic approach from the government about what is possible. Instead of constantly beating GP's alongside poor investment in primary care, looking at what we do and how well we do it should be championed. The BMA need to do more actively to support the profession and the RCGP too…. It is not acceptable to raise expectations of the public saying we can open all hours and weekends. If the money is not there to pay us and to have enough manpower in the practice then they can't have the service”* (ID 79, 40–49, female, principal).

*“We need to be responsible for half the number of patients per doctor, i.e. we need double the number of GPs, now that we manage increasingly complex medical problems, carrying out much more intensive chronic disease management, in an increasingly frail and elderly population”* (ID 344, 50–59, female, principal).

*“If the government wants to improve GP retention and recruitment then it will need to greatly reduce the bureaucracy and administrative burden on general practice and properly fund additional clinical staff”* (95, age 40–49, male, principal).

*“Less meaningless bureaucracy is the main factor that would improve my working life. Much has been suggested to help reduce the bureaucracy of the job - little has been delivered”* (ID 395, age 50–59, female, principal).

Remuneration and pay also emerged as important issues for many GPs:

*“The fact that pay has effectively stalled over the last 10 year combined with the additional superann …effectively this is just more tax as we don't get any more out of the NHS pension scheme for putting more in…”*(ID 378, age 50–59, male, principal).

*“Personally I don't want increased pay I'd rather have manageable workload BUT I'm aware that diminishing remuneration is having an impact on my younger colleagues' motivation to stay a partner and impacting ability to recruit quality GPs”* (ID 161, age 50–59, female, principal).

In addition, addressing the costs of indemnity and professional registration was seen as important:

*“Indemnity fees are shocking. Introduction of government compensation scheme like those in Australia and New Zealand would strongly encourage me to continue”* (ID 390, age 40–49, female, freelance GP).

The need for more supportive structures and networks was also emphasised, together with a more flexible and developmental approach to GP appraisal and revalidation:

*“Local support for struggling GPs i.e. access to counselling and CBT locally, face to face and confidential. RCGP should stop adding hoops to GMC revalidation supporting information requirements. All GP's should have access to a network not just '20 plus' and 'first 5'. Do not forget the 1 in 4 GP's who are locums. Allow GP's to take career breaks and fund/support their return to general practice”* (ID 390, age 40–49, female, freelance GP).

*“Flexible criteria for appraisals and revalidation to suit doctors with different levels of experience. One size fits all process should be stopped”* (ID 418, age 30–39, female, practice-employed salaried).

## Discussion

This study indicates the breadth and magnitude of factors contributing to the workforce crisis facing general practice in England. The scale of this crisis may be even greater than previously reported, with 82.0 % participants in this study stating that they intend to leave general practice, take a career break or reduce their clinical hours within the next five years; in contrast, only 5.6 % plan to increase their hours. The statistical and qualitative analyses go further than previous surveys to draw attention to the number and nature of the features that are contributing to this crisis, and the cumulative effect that these are having on individual practitioners at different stages of the career path. Workplace influences, such as the intensity and volume of workload, the increasing expectations being placed on general practice by government, the NHS and patients, the erosion of autonomy, increasing hours of work and the difficulties recruiting and retaining GPs emerge as key issues fuelling the crisis, resulting in low morale, distress, diminished job satisfaction and expressed intentions to retire, take a career break or reduce hours.

Four principal components were identified that are strongly associated with intentions to leave general practice, and as summarised in Table 3 they are differentially associated with specific GP attributes. Although previous research has described the ways in which salaried (ie non-principal) GPs feel disenfranchised and disillusioned by their role [17], we found that being a GP principal was more likely to be associated with the principal components “overall workload” and “personal development” intentions. While expressed intentions to retire or take a career break may not directly translate into action, the intention to leave has been shown to be an accurate predictor of subsequent actions [18]. The qualitative analysis revealed how workload and other work-related pressures, exacerbated by critical government and media attention, have created an emotionally and physically exhausted workforce that many GPs feel increasingly unable or unwilling to endure.

**Table 3:** Summary of GP attributes associated with the principal components linked to intention to leave general practice within the next 5 years

Principal Component	Associated GP attributes
	(likelihood ratio test significance)
Overall workload	- Age < 50 years (*p* < 0.001)
	- GP principal (*p* < 0.001)
Working conditions	- Age < 50 years (*p* < 0.001)
	- Male (*p* < 0.001)
	- GP appraiser (*p* < 0.001)
	- Smaller practice size (*p* < 0.001)
Work-life flexibility	- Age < 50 years (*p* = 0.002)
	- Female (*p* = 0.002)
	- Fewer years in practice (*p* < 0.001)
	- Smaller practice size (*p* = 0.006)
	- Portfolio career GP (*p* = 0.02)
Personal development	- Female (*p* = 0.013)
	- >10 years in service (*p* = 0.012)
	- GP principal (*p* = 0.029)

### Strengths and weaknesses of the study

A major strength is the diversity of GPs that participated, from those early in their career and undertaking portfolio careers to those approaching retirement, from practices of all sizes, and from settings ranging from inner city to rural. A further strength is that our study explored not only intentions to leave general practice or reduce working hours, but also to take a career break. We not only explored the factors that underlie both, but identified factors which might encourage GPs to remain in the workforce. The mixed methods approach using statistical and qualitative analyses provided a more fine-grained assessment of the scale and depth of GPs’ concerns and feelings than has emerged from studies limited to one or other methodology.

A key limitation is the uncertainty over the response rate. Owing to governance issues, we faced difficulties in obtaining GPs’ contact details and hence relied on emailed invitations to practices being forwarded by managers to potential participants, and that the intended recipients read the email. The survey was undertaken around the Christmas/New Year period and seasonal factors, such as workload pressures prevalent at this time of year, may have influenced responses. It is possible that those who felt most over-worked or disillusioned may have been less likely to notice or feel motivated to respond to the invitation to the survey. However, support for the representativeness of our sample comes from their characteristics being broadly similar to those from the region who responded to the recent national BMA survey [10]. In addition, we controlled in the data analysis for the over-representation of participants aged between 50–59 years, and of UK/Ireland-trained GPs compared to the regional population of GPs. While the findings need to be interpreted with some caution as they relate to a single region in England, the BMA survey found little variation across regions which means that the findings are likely to be applicable across all areas [10].

It was beyond the scope of the questionnaire to consider contextual factors, such as the composition of the general practice workforce composition, appointment and triage systems and the working environment within the practice. While general practice nurses and allied health professionals (AHPs) are key to the delivery of modern general practice, there is a lack of evidence about the impact that doctor-nurse/AHP substitution has on doctors' workload [19]. However, it was interesting that difficulties in recruiting and retaining the wider primary care workforce did not emerge as a theme in the qualitative analysis, and hence from this data does not appear to be an important issue affecting GPs’ intentions to leave practice.

## Conclusions

Key factors that lead to professional burnout include work overload, lack of control, insufficient reward and value conflict [20]. The findings from our study provide evidence of how GPs across the career path are experiencing such factors, and are in line with those from a recent cross-sectional study of GPs which found high levels of emotional exhaustion (46 % respondents), feelings of depersonalisation (42 %) and low personal accomplishment (34 %) [21].

There is an urgent need to tackle these issues in order to prevent burnout and to encourage GPs to develop coping strategies and interests that may have a protective effect. Promoting job satisfaction and morale may not only help to retain the current workforce, but will also make general practice a more attractive career option for medical students and recently qualified doctors [22]. A coordinated approach is required between government and the NHS to address these issues, and in particular reduce workload intensity, volume and administrative demands, with greater time for patient care and increased clinical autonomy [7, 23].

The recently published Royal College of General Practitioners strategy “A blueprint for building the new deal for general practice in England” [24] articulates a way forward; however, it does not sufficiently address the immediate and pressing concerns that GPs expressed in this study. A multi-facetted approach is needed to address the findings presented here. This ought to include the following types of measure: an urgent review of the administrative burden on general practice and GPs aimed at minimising and/or reallocating such work; specific interventions aimed at supporting portfolio careers, and addressing barriers to engagement; analysis of workload that is being transferred from secondary to primary care to identify what is needed, what can be re-allocated, what can be avoided; research to better understand the impact on GP demand and workload of the changes in case mix, severity and complexity encountered in everyday consultations; redesigning appraisal and revalidation in order to support GP re-engagement with professionalism; increased psychological support and career guidance for GPs; engaging with the media and more honest public debate about expectations and the GP role.

In conclusion, the findings from this survey provide evidence of an immediate need for measures to improve GPs’ working lives. While many of the findings reinforce previous work, such as GPs' experience of the intensity and volume of workload, there were several new issues that emerged reflecting ways in which the experience of working as a GP has deteriorated, such as the impact of the substantial transfer of unfunded work from secondary care without commensurate funding or resources, time spent on tasks that are perceived as unimportant, changes to pensions and the introduction of seven-day working. Further research is required to understand more fully the impact of these factors on GPs’ career plans, as well as the impact of greater practice nurse/health care assistant/physician assistant workforce mix on GPs’ workload and experience. New ways to improve the working conditions of the GP workforce are needed, including measures that are sensitive to the needs of GPs at difference stages of the career path; for example, the importance of personal development opportunities for female GPs, GPs with over 10 years' service and GP principals.

These findings have clear policy implications, especially the need to address the emotional impact of general practice workload and to develop the organisation and skill mix of the general practice workforce to more effectively and sustainably deliver care. Initiatives such as “Building the workforce - the New Deal for GPs” appear insufficient [11]. New models of professionalism and organisational structure, including further development of the multi-disciplinary general practice team, are needed to revitalise the GP workforce and so create a sustainable career in which GPs are better able to cope with the hours of work, workload and administrative activities.

## Additional file

Additional file 1:
**The following supplementary tables are available:** **Table S1.** Participant demographics and practice characteristics. **Table S2.** Logistic regression analysis (a) intention to leave practice within 5 years (b) and intention to take a career break within 5 years. **Table S3.** Factors influencing GPs’ decision to retire/leave practice: (a) work related (b) non-work related. **Table S4.** Factors that might retain GPs in practice: (a) work-related and (b) non-work related. In addition, a statement summarising adherence to the COREQ guidelines is also provided. (DOCX 31 kb)

## Abbreviations

CCG: Clinical Commissioning Group
CI: Confidence Interval
CQC: Care Quality Commission
GMC: General Medical Council
GSF: Gold Standards Framework
HV: Health Visitor
LRT: Likelihood Ratio Test
NHS: National Health Service
OOH: Out of hours
PC: Principal Component
PCA: Principal Components Analysis
RCGP: Royal College of General Practitioners
