# Supplementary material for: Retaining the general practitioner workforce in England: what matters to GPs? A cross-sectional study
Source: BMC Fam Pract. 2015 Oct 16;16:140. doi: 10.1186/s12875-015-0363-1 (PMC4608111; doi:10.1186/s12875-015-0363-1)
Supplement: Additional file 1: — The following supplementary tables are available: Table S1. Participant demographics and practice characteristics. Table S2. Logistic regression analysis (a) intention to leave practice within 5 years (b) and intention to take a career break within 5 years. Table S3. Factors influencing GPs’ decision to retire/leave practice: (a) work related (b) non-work related. Table S4. Factors that might retain GPs in practice: (a) work-related and (b) non-work related. In addition, a statement summarising adherence to the COREQ guidelines is also provided. (DOCX 31 kb) [file 12875_2015_363_MOESM1_ESM.docx]

**SUPPLEMENTARY TABLES**

**Table S1. Participant demographics and practice characteristics**

|  | Study Participants | All GPs West Midlands |
| --- | --- | --- |
| Number | **1192** | **3686** |
| Gender  Male  Female | **Frequency (percent)**  622 (54.7%)  515 (45.3%) | **Frequency (percent)**  2050 (55.7%)  1633 (44.3%) |
| Age (years)  25-29 years  30-39 years  40-49 years  50-59 years  60-69 years  70 or more years | 14 (1.2%)  263 (23.0%)  338 (29.6%)  436 (38.2%)  84 (7.4%)  7 (0.6%) | 55 (1.5%)  1043 (28%)  1089 (29.6%)  1043 (28.4%)  371 (10.1%)  77 (2.1%) |
| Place of qualification  UK and Ireland  Rest of Europe  Other | 959 (85.8%)  32 (2.9%)  127 (11.4%) | 2805 (72.6%)  142 (3.7%)  913 (23.6%) |
| Main employment status  GP contractor/principal  Practice-employed salaried GP  NHS trust-employed salaried GP  Private sector-employed salaried GP  Freelance GP (locum)  Out-of-hours GP  Academic GP | 876 (74.9%)  212 (18.1%)  12 (1.0%)  2 (0.2%)  56 (4.8%)  8 (0.7%)  4 (0.3%) | 2797 (72.4%) |
| Additional roles (current)  CCG role  LMC role  Appraiser  GP trainer  Undergraduate student tutor  Postgraduate tutor-other educationalist  Research  Hospital based clinical assistant  Community based clinical assistant  GP with specialist interest | 169 (14.2%)  101 (8.5%)  160 (13.4)  338 (28.4)  245 (20.6%)  105 (8.8%)  96 (8.1%)  50 (4.2%)  24 (2.0%)  232 (19.5%) |  |
| Number of additional roles  No additional role (s)  One additional role  More than one additional role | 420 (35.2%)  335 (28.1%)  437 (36.7%) |  |
| Length of time in general practice  Less than 5 years  5 - 9 years  10 - 19 years  20 - 29 years  30 or more years | 106 (9.0%)  193 (16.3%)  314 (26.6%)  421 (35.6%)  147 (12.4%) |  |
| Numbers of hours worked  Up to 10 hours  11-20 hours  21-30 hours  31-40 hours  41 or more hours | 33 (2.8%)  72 (6.2%)  195 (16.7%)  285 (24.4%)  582 (49.9%) |  |
| Practice list size  Less than 4,000  4,000 – 9,999  10,000 – 14,999  15,000 or more | 108 (9.5%)  510 (45.1%)  379 (33.5%)  134 (11.8%) |  |
| Number of GPs in practice  1 - 2  3- 5  6-10  11 or more | 95 (8.4%)  395 (35.0%)  568 (50.3%)  72 (6.4%) | 36.5% |
| Location  Inner city  Other urban  Urban/rural mix  Rural/isolated rural | 200 (17.6%)  432 (38.0%)  364 (32.0%)  140 (12.3%) |  |

**Table S2. Logistic regression analysis (a) intention to leave practice within 5 years (b) and intention to take a career break within 5 years**

| ***(a) intention to leave practice within 5 years*** | | | | | | | | |
| --- | --- | --- | --- | --- | --- | --- | --- | --- |
| Regression Coefficient Estimates: | | | | | | | | |
| Coefficients | | | Estimate | | Std. Error | | Pr(>\|z\|) | |
| Intercept | | | -1.5008 | | 0.2459 | | 1.04e-09 | *** |
| Age 50+ | | | 2.2175 | | 0.2218 | | < 2e-16 | *** |
| Gender F | | | -1.0060 | | 0.2635 | | 0.000135 | *** |
| Length of service 10+ | | | 0.7406 | | 0.2508 | | 0.003142 | ** |
| Additional Roles 1+ | | | -0.5755 | | 0.1806 | | 0.001437 | ** |
| Age 50+ : Gender F | | | 0.9456 | | 0.3445 | | 0.006058 | ** |
| Null deviance: 1387.99 on 1026 degrees of freedom; Residual deviance: 952.21 on 1021 degrees of freedom; AIC: 964.21 | | | | | | | | |
| Single term deletions: | | | | | | | | |
|  | Df | Deviance | | AIC | | LRT | Pr(>Chi) | |
| <none> |  | 952.21 | | 964.21 | |  |  | |
| Length of service 10+ | 1 | 961.25 | | 971.25 | | 9.0340 | 0.002650 | ** |
| Additional Roles 1+ | 1 | 962.62 | | 972.62 | | 10.4091 | 0.001254 | ** |
| Age 50+ : Gender F | 1 | 959.95 | | 969.95 | | 7.7353 | 0.005415 | ** |
| Significance codes: 0 ‘***’ 0.001 ‘**’ 0.01 ‘*’ 0.05 ‘.’ 0.1 ‘ ’ 1 | | | | | | | | |

| ***(b) intention to take a career break within 5 years*** | | | | | | | | |
| --- | --- | --- | --- | --- | --- | --- | --- | --- |
| Regression Coefficient Estimates: | | | | | | | | |
| Coefficients | | | Estimate | | Std. Error | | Pr(>\|z\|) | |
| *Intercept* | | | *-1.1683* | | *0.1238* | | *< 2e-16* | ***** |
| *Age 50+* | | | *-0.4801* | | *0.1510* | | *0.00147* | **** |
| *Hours worked 40+* | | | *0.4044* | | *0.1490* | | *0.00665* | **** |
| *Null deviance: 1127.9 on 1025 degrees of freedom; Residual deviance: 1111.2 on 1023 degrees of freedom; AIC: 1117.2* | | | | | | | | |
| Single term deletions: | | | | | | | | |
|  | Df | Deviance | | AIC | | LRT | Pr(>Chi) | |
| <none> |  | *1111.2* | | *1117.2* | |  |  | |
| Age | 1 | *1121.5* | | *1125.5* | | *10.3128* | *0.001321* | **** |
| Hours worked | 1 | *1118.6* | | *1122.6* | | *7.4436* | *0.006366* | **** |
| Significance codes: 0 ‘***’ 0.001 ‘**’ 0.01 ‘*’ 0.05 ‘.’ 0.1 ‘ ’ 1 | | | | | | | | |

**Table S3. Factors influencing GPs’ decision to retire/leave practice: (a) work related (b) non-work related**

| 1. ***Work Related Factors***: | | | | | | | | | | | | | | | | | | | | | | | |
| --- | --- | --- | --- | --- | --- | --- | --- | --- | --- | --- | --- | --- | --- | --- | --- | --- | --- | --- | --- | --- | --- | --- | --- |
| *a. Volume of workload* | | | | | | | | | | | | | | | | | | | | | | | |
| *b. Intensity of workload* | | | | | | | | | | | | | | | | | | | | | | | |
| *c. Lack of time for patient contact* | | | | | | | | | | | | | | | | | | | | | | | |
| *d. Too much time spent on unimportant tasks* | | | | | | | | | | | | | | | | | | | | | | | |
| *e. Poor flexibility of hours* | | | | | | | | | | | | | | | | | | | | | | | |
| *f. Potential introduction of 7 day a week working* | | | | | | | | | | | | | | | | | | | | | | | |
| *g. Reduced job satisfaction* | | | | | | | | | | | | | | | | | | | | | | | |
| *h. Revalidation* | | | | | | | | | | | | | | | | | | | | | | | |
| *i. Changes to pension taxation* | | | | | | | | | | | | | | | | | | | | | | | |
| PCA: | | | | | | | | | | | | | | | | | | | | | | | |
| Principal Components: | PC1 | | | PC2 | | PC3 | | | PC4 | | | PC5 | | | PC6 | | | PC7 | | PC8 | | PC9 | |
| Standard deviation | 1.833 | | | 1.151 | | 0.958 | | | 0.932 | | | 0.866 | | | 0.797 | | | 0.747 | | 0.664 | | 0.376 | |
| Proportion of Variance | 0.373 | | | 0.147 | | 0.102 | | | 0.097 | | | 0.083 | | | 0.071 | | | 0.062 | | 0.049 | | 0.016 | |
| Cumulative Proportion | 0.373 | | | 0.521 | | 0.623 | | | 0.719 | | | 0.803 | | | 0.873 | | | 0.935 | | 0.984 | | 1.000 | |
| Loadings: | a. | | | b. | | c. | | | d. | | | e. | | | f. | | | g. | | h. | | i. | |
| PC1: *Overall workload* | -0.42 | | | -0.40 | | -0.34 | | | -0.37 | | | -0.29 | | | -0.35 | | | -0.37 | | -0.12 | | -0.23 | |
| PC2: *Working conditions* | 0.38 | | | 0.42 | | 0.08 | | | -0.07 | | | -0.27 | | | -0.15 | | | -0.06 | | -0.61 | | -0.44 | |
| PC1 Regression Coefficient Estimates: | | | | | | | | | | | | | | | | | | | | | | | |
| Coefficients | | | | | Estimate | | | | | | Std. Error | | | | | Pr(>\|z\|) | | | | | | | |
| Intercept | | | | | -0.9401 | | | | | | 0.2309 | | | | | 5.67e-05 | | | | | | *** | |
| Age 50+ | | | | | 0.9452 | | | | | | 0.2442 | | | | | 0.000127 | | | | | | *** | |
| GP Role Other | | | | | 1.2758 | | | | | | 0.2682 | | | | | 2.78e-06 | | | | | | *** | |
| Residual standard error: 1.82 on 388 degrees of freedom; Multiple R-squared: 0.07328, Adjusted R-squared: 0.0685; F-statistic: 15.34 on 2 and 388 DF, p-value: 3.874e-07 | | | | | | | | | | | | | | | | | | | | | | | |
| Single term deletions: | | | | | | | | | | | | | | | | | | | | | | | |
|  | | Df | Sum of Sq | | | | | RSS | | | | | AIC | | | | Pr(>Chi) | | | | | | |
| <none> | |  |  | | | | | 1284.6 | | | | | 471.09 | | | |  | | | | | | |
| Age | | 1 | 49.609 | | | | | 1334.2 | | | | | 483.90 | | | | 0.0001185 | | | | | | *** |
| GP Role | | 1 | 74.921 | | | | | 1359.5 | | | | | 491.25 | | | | 2.503e-06 | | | | | | *** |
| Signif. codes: 0 ‘***’ 0.001 ‘**’ 0.01 ‘*’ 0.05 ‘.’ 0.1 ‘ ’ 1 | | | | | | | | | | | | | | | | | | | | | | | |
| PC2 Regression Coefficient Estimates: | | | | | | | | | | | | | | | | | | | | | | | |
| Coefficients | | | | | Estimate | | | | | | Std. Error | | | | | Pr(>\|z\|) | | | | | | | |
| Intercept | | | | | -0.7154 | | | | | | 0.2372 | | | | | 0.002727 | | | | | | ** | |
| Age 50+ | | | | | 0.5318 | | | | | | 0.1354 | | | | | 0.000102 | | | | | | *** | |
| Gender F | | | | | 0.6038 | | | | | | 0.1135 | | | | | 1.76e-07 | | | | | | *** | |
| GPs 3-5 | | | | | 0.5150 | | | | | | 0.1819 | | | | | 0.004884 | | | | | | ** | |
| GPs 6-10 | | | | | 0.7416 | | | | | | 0.1794 | | | | | 4.37e-05 | | | | | | *** | |
| GPs >11 | | | | | 0.6877 | | | | | | 0.2818 | | | | | 0.015129 | | | | | | * | |
| Appraiser N | | | | | -0.5964 | | | | | | 0.1455 | | | | | 5.08e-05 | | | | | | *** | |
| Residual standard error: 1.032 on 384 degrees of freedom; Multiple R-squared: 0.1853, Adjusted R-squared: 0.1726; F-statistic: 14.56 on 6 and 384 DF, p-value: 5.472e-15 | | | | | | | | | | | | | | | | | | | | | | | |
| Single term deletions: | | | | | | | | | | | | | | | | | | | | | | | |
|  | | Df | Sum of Sq | | | | | RSS | | | | | AIC | | | | Pr(>Chi) | | | | | | |
| <none> | |  |  | | | | | 408.65 | | | | | 31.264 | | | |  | | | | | | |
| Age | | 1 | 16.419 | | | | | 425.07 | | | | | 44.667 | | | | 8.686e-05 | | | | | | *** |
| Gender | | 1 | 30.123 | | | | | 438.77 | | | | | 57.074 | | | | 1.339e-07 | | | | | | *** |
| GPs | | 3 | 19.028 | | | | | 427.68 | | | | | 43.059 | | | | 0.0004848 | | | | | | *** |
| Appraiser | | 1 | 17.876 | | | | | 426.53 | | | | | 46.004 | | | | 4.287e-05 | | | | | | *** |
| Significance codes: 0 ‘***’ 0.001 ‘**’ 0.01 ‘*’ 0.05 ‘.’ 0.1 ‘ ’ 1 | | | | | | | | | | | | | | | | | | | | | | | |
|  | | | | | | | | | | | | | | | | | | | | | | | |
| 1. ***Non-work Related Factors:*** | | | | | | | | | | | | | | | | | | | | | | | |
| *j. Age* | | | | | | | | | | | | | | | | | | | | | | | |
| *k. Family commitments* | | | | | | | | | | | | | | | | | | | | | | | |
| *l. Ill health* | | | | | | | | | | | | | | | | | | | | | | | |
| *m. Embarking on career outside general practice* | | | | | | | | | | | | | | | | | | | | | | | |
| *n. Planned career break* | | | | | | | | | | | | | | | | | | | | | | | |
| PCA: | | | | | | | PC1 | | | PC2 | | | | PC3 | | | | | PC4 | | PC5 | | |
| Principal Components: | | | | | | | 1.351 | | | 1.167 | | | | 0.857 | | | | | 0.776 | | 0.690 | | |
| Standard deviation | | | | | | | 0.365 | | | 0.272 | | | | 0.147 | | | | | 0.120 | | 0.095 | | |
| Proportion of Variance | | | | | | | 0.365 | | | 0.637 | | | | 0.784 | | | | | 0.905 | | 1.000 | | |
| Cumulative Proportion | | | | | | | j. | | | k. | | | | l. | | | | | m. | | n. | | |
| Loadings: | | | | | | | 0.24 | | | 0.46 | | | | 0.47 | | | | | 0.47 | | 0.53 | | |
| PC1: *Work-life balance* | | | | | | | -0.66 | | | -0.38 | | | | -0.21 | | | | | 0.49 | | 0.37 | | |
| PC2: *Personal development* | | | | | | | | | | | | | | | | | | | | | | | |
| PC1 Regression Coefficient Estimates: | | | | | | | | | | | | | | | | | | | | | | | |
| Coefficients | | | | | Estimate | | | | | | Std. Error | | | | | Pr(>\|z\|) | | | | | | | |
| Intercept | | | | | 0.4722 | | | | | | 0.1697 | | | | | 0.005682 | | | | | | ** | |
| Age 50+ | | | | | -0.6966 | | | | | | 0.1803 | | | | | 0.000132 | | | | | | *** | |
| GP Role Other | | | | | 0.5870 | | | | | | 0.2071 | | | | | 0.004841 | | | | | | ** | |
| Residual standard error: 1.327 on 369 degrees of freedom; Multiple R-squared: 0.07688, Adjusted R-squared: 0.07187; F-statistic: 15.37 on 2 and 369 DF, p-value: 3.893e-07 | | | | | | | | | | | | | | | | | | | | | | | |
| Single term deletions: | | | | | | | | | | | | | | | | | | | | | | | |
|  | | Df | Sum of Sq | | | | | RSS | | | | | AIC | | | | Pr(>Chi) | | | | | | |
| <none> | |  |  | | | | | 649.31 | | | | | 213.21 | | | |  | | | | | | |
| Age | | 1 | 26.276 | | | | | 675.58 | | | | | 225.97 | | | | 0.0001223 | | | | | | *** |
| GP Role | | 1 | 14.139 | | | | | 663.44 | | | | | 219.22 | | | | 0.0046434 | | | | | | ** |
| Significance codes: 0 ‘***’ 0.001 ‘**’ 0.01 ‘*’ 0.05 ‘.’ 0.1 ‘ ’ 1 | | | | | | | | | | | | | | | | | | | | | | | |
| PC2 Regression Coefficient Estimates: | | | | | | | | | | | | | | | | | | | | | | | |
| Coefficients | | | | | Estimate | | | | | | Std. Error | | | | | Pr(>\|z\|) | | | | | | | |
| Intercept | | | | | 1.1256 | | | | | | 0.1242 | | | | | < 2e-16 | | | | | | *** | |
| Age 50+ | | | | | -1.3032 | | | | | | 0.1367 | | | | | < 2e-16 | | | | | | *** | |
| Additional Roles 1+ | | | | | -0.3212 | | | | | | 0.1180 | | | | | 0.00682 | | | | | | ** | |
| Residual standard error: 1.035 on 369 degrees of freedom; Multiple R-squared: 0.2221, Adjusted R-squared: 0.2179; F-statistic: 52.67 on 2 and 369 DF, p-value: < 2.2e-16 | | | | | | | | | | | | | | | | | | | | | | | |
| Single term deletions: | | | | | | | | | | | | | | | | | | | | | | | |
|  | | Df | Sum of Sq | | | | | RSS | | | | | AIC | | | | Pr(>Chi) | | | | | | |
| <none> | |  |  | | | | | 395.50 | | | | | 28.792 | | | |  | | | | | | |
| Age | | 1 | 97.391 | | | | | 492.90 | | | | | 108.682 | | | | < 2e-16 | | | | | | *** |
| Additional Role | | 1 | 7.935 | | | | | 403.44 | | | | | 34.182 | | | | 0.00656 | | | | | | ** |
| Significance codes: 0 ‘***’ 0.001 ‘**’ 0.01 ‘*’ 0.05 ‘.’ 0.1 ‘ ’ 1 | | | | | | | | | | | | | | | | | | | | | | | |

**Table S4. Factors that might retain GPs in practice: (a) work-related and (b) non-work related**

| 1. ***Work Related Factors:*** | | | | | | | | | | | | | | | | | | | | | | | | |
| --- | --- | --- | --- | --- | --- | --- | --- | --- | --- | --- | --- | --- | --- | --- | --- | --- | --- | --- | --- | --- | --- | --- | --- | --- |
| a. Reduced volume of workload | | | | | | | | | | | | | | | | | | | | | | | | |
| b. Reduced intensity of workload | | | | | | | | | | | | | | | | | | | | | | | | |
| c. More flexible working conditions | | | | | | | | | | | | | | | | | | | | | | | | |
| d. Longer appointment times/more time to spend with patients | | | | | | | | | | | | | | | | | | | | | | | | |
| e. Improved skill-mix in the practice | | | | | | | | | | | | | | | | | | | | | | | | |
| f. Shorter practice opening times | | | | | | | | | | | | | | | | | | | | | | | | |
| g. Less administration | | | | | | | | | | | | | | | | | | | | | | | | |
| h. No out of hours commitments | | | | | | | | | | | | | | | | | | | | | | | | |
| PCA: | | | | | | | | | | | | | | | | | | | | | | | | |
| Principal Components: | | | PC1 | | PC2 | | | PC3 | | | PC4 | | | PC5 | | | | PC6 | | PC7 | | | PC8 | |
| Standard deviation | | | 1.912 | | 1.043 | | | 0.944 | | | 0.800 | | | 0.778 | | | | 0.754 | | 0.644 | | | 0.371 | |
| Proportion of Variance | | | 0.457 | | 0.136 | | | 0.111 | | | 0.080 | | | 0.076 | | | | 0.071 | | 0.052 | | | 0.017 | |
| Cumulative Proportion | | | 0.457 | | 0.593 | | | 0.704 | | | 0.784 | | | 0.860 | | | | 0.931 | | 0.983 | | | 1.000 | |
| Loadings: | | | a. | | b. | | | c. | | | d. | | | e. | | | | f. | | g. | | | h. | |
| PC1: *Overall workload* | | | -0.42 | | -0.42 | | | -0.35 | | | -0.35 | | | -0.34 | | | | -0.31 | | -0.33 | | | -0.29 | |
| PC2: *Working conditions* | | | 0.44 | | 0.45 | | | -0.33 | | | 0.19 | | | -0.41 | | | | -0.44 | | 0.11 | | | -0.30 | |
| PC1 Regression Coefficient Estimates: | | | | | | | | | | | | | | | | | | | | | | | |  |
| Coefficients | | | | Estimate | | | | | | Std. Error | | | | | | Pr(>\|z\|) | | | | | | | |  |
| Intercept | | | | -0.4326 | | | | | | 0.2259 | | | | | | 0.0562 | | | | | | . | |  |
| Age 50+ | | | | 0.5707 | | | | | | 0.2509 | | | | | | 0.0235 | | | | | | * | |  |
| Residual standard error: 1.903 on 373 degrees of freedom; Multiple R-squared: 0.01369, Adjusted R-squared: 0.01104; F-statistic: 5.176 on 1 and 373 DF, p-value: 0.02347 | | | | | | | | | | | | | | | | | | | | | | | |  |
| Single term deletions: | | | | | | | | | | | | | | | | | | | | | | | |  |
|  | Df | Sum of Sq | | | | | RSS | | | | | | AIC | | | | Pr(>Chi) | | | | | | |  |
| <none> |  |  | | | | | 1351.0 | | | | | | 484.63 | | | |  | | | | | | |  |
| Age | 1 | 18.746 | | | | | 1369.8 | | | | | | 487.80 | | | | 0.02301 | | | | | | * |  |
| Significance codes: 0 ‘***’ 0.001 ‘**’ 0.01 ‘*’ 0.05 ‘.’ 0.1 ‘ ’ 1 | | | | | | | | | | | | | | | | | | | | | | | |  |
| PC2 Regression Coefficient Estimates: | | | | | | | | | | | | | | | | | | | | | | | |  |
| Coefficients | | | | Estimate | | | | | | Std. Error | | | | | | Pr(>\|z\|) | | | | | | | |  |
| Intercept | | | | -0.3284 | | | | | | 0.1236 | | | | | | 0.00823 | | | | | | ** | |  |
| Age 50+ | | | | 0.4239 | | | | | | 0.1373 | | | | | | 0.00217 | | | | | | ** | |  |
| Residual standard error: 1.041 on 373 degrees of freedom; Multiple R-squared: 0.02492, Adjusted R-squared: 0.02231; F-statistic: 9.535 on 1 and 373 DF, p-value: 0.002167 | | | | | | | | | | | | | | | | | | | | | | | |  |
| Single term deletions: | | | | | | | | | | | | | | | | | | | | | | | |  |
|  | Df | Sum of Sq | | | | | RSS | | | | | | AIC | | | | Pr(>Chi) | | | | | | |  |
| <none> |  |  | | | | | 404.55 | | | | | | 32.443 | | | |  | | | | | | |  |
| Age | 1 | 10.341 | | | | | 414.89 | | | | | | 39.908 | | | | 0.002094 | | | | | | ** |  |
| Significance codes: 0 ‘***’ 0.001 ‘**’ 0.01 ‘*’ 0.05 ‘.’ 0.1 ‘ ’ 1 | | | | | | | | | | | | | | | | | | | | | | | |  |
|  | | | | | | | | | | | | | | | | | | | | | | | | |
| 1. ***Non-work Related Factors:*** | | | | | | | | | | | | | | | | | | | | | | | | |
| i. Option to work term time only | | | | | | | | | | | | | | | | | | | | | | | | |
| j. Greater clinical autonomy | | | | | | | | | | | | | | | | | | | | | | | | |
| k. Additional annual leave | | | | | | | | | | | | | | | | | | | | | | | | |
| l. Opportunity for a sabbatical | | | | | | | | | | | | | | | | | | | | | | | | |
| m. Protected time for education and training | | | | | | | | | | | | | | | | | | | | | | | | |
| n. Reintroduction of the flexible careers scheme | | | | | | | | | | | | | | | | | | | | | | | | |
| o. Expansion of GP retainer scheme | | | | | | | | | | | | | | | | | | | | | | | | |
| PCA: | | | | | | | | | | | | | | | | | | | | | | | | |
| Principal Components: | | | PC1 | | | PC2 | | | PC3 | | | PC4 | | | PC5 | | | | PC6 | | PC7 | | | |
| Standard deviation | | | 1.818 | | | 0.977 | | | 0.890 | | | 0.852 | | | 0.751 | | | | 0.615 | | 0.530 | | | |
| Proportion of Variance | | | 0.472 | | | 0.136 | | | 0.113 | | | 0.104 | | | 0.080 | | | | 0.054 | | 0.040 | | | |
| Cumulative Proportion | | | 0.472 | | | 0.609 | | | 0.722 | | | 0.825 | | | 0.906 | | | | 0.960 | | 1.000 | | | |
| Loadings: | | | i. | | | j. | | | k. | | | l. | | | m. | | | | n. | | o. | | | |
| PC1: W*ork flexibility* | | | 0.31 | | | 0.30 | | | 0.39 | | | 0.42 | | | 0.36 | | | | 0.43 | | 0.41 | | | |
| PC2: *Training opportunities* | | | -0.44 | | | 0.45 | | | 0.18 | | | 0.21 | | | 0.48 | | | | -0.34 | | -0.43 | | | |
| PC1 Regression Coefficient Estimates: | | | | | | | | | | | | | | | | | | | | | | | |  |
| Coefficients | | | | Estimate | | | | | | Std. Error | | | | | | Pr(>\|z\|) | | | | | | | |  |
| Intercept | | | | 1.9729 | | | | | | 0.4119 | | | | | | 2.44e-06 | | | | | | *** | |  |
| Age 50+ | | | | -0.8130 | | | | | | 0.2634 | | | | | | 0.00218 | | | | | | ** | |  |
| Gender F | | | | 0.6014 | | | | | | 0.1902 | | | | | | 0.00170 | | | | | | ** | |  |
| GPs 3-5 | | | | -0.5898 | | | | | | 0.3046 | | | | | | 0.05357 | | | | | | . | |  |
| GPs 6-10 | | | | -0.9855 | | | | | | 0.2999 | | | | | | 0.00111 | | | | | | ** | |  |
| GPs >11 | | | | -1.0725 | | | | | | 0.4878 | | | | | | 0.02853 | | | | | | * | |  |
| Length of service 10+ | | | | -1.2349 | | | | | | 0.3832 | | | | | | 0.00139 | | | | | | ** | |  |
| Add Role 1+ | | | | 0.4379 | | | | | | 0.1901 | | | | | | 0.02179 | | | | | | * | |  |
| Residual standard error: 1.693 on 363 degrees of freedom; Multiple R-squared: 0.1538, Adjusted R-squared: 0.1375; F-statistic: 9.424 on 7 and 363 DF, p-value: 9.426e-11 | | | | | | | | | | | | | | | | | | | | | | | |  |
| Single term deletions: | | | | | | | | | | | | | | | | | | | | | | | |  |
|  | Df | Sum of Sq | | | | | RSS | | | | | | AIC | | | | Pr(>Chi) | | | | | | |  |
| <none> |  |  | | | | | 1040.8 | | | | | | 398.68 | | | |  | | | | | | |  |
| Age | 1 | 27.304 | | | | | 1068.0 | | | | | | 406.29 | | | | 0.001938 | | | | | | ** |  |
| Gender | 1 | 28.655 | | | | | 1069.4 | | | | | | 406.76 | | | | 0.001501 | | | | | | ** |  |
| GPs | 3 | 36.023 | | | | | 1076.8 | | | | | | 405.31 | | | | 0.005524 | | | | | | ** |  |
| Length of service | 1 | 29.773 | | | | | 1070.5 | | | | | | 407.15 | | | | 0.001217 | | | | | | ** |  |
| Additional Role | 1 | 15.218 | | | | | 1056.0 | | | | | | 402.07 | | | | 0.020304 | | | | | | * |  |
| Significance codes: 0 ‘***’ 0.001 ‘**’ 0.01 ‘*’ 0.05 ‘.’ 0.1 ‘ ’ 1 | | | | | | | | | | | | | | | | | | | | | | | |  |
| PC2 Regression Coefficient Estimates: | | | | | | | | | | | | | | | | | | | | | | | |  |
| Coefficients | | | | Estimate | | | | | | Std. Error | | | | | | Pr(>\|z\|) | | | | | | | |  |
| Intercept | | | | -0.4338 | | | | | | 0.1866 | | | | | | 0.0206 | | | | | | * | |  |
| Gender F | | | | 0.2591 | | | | | | 0.1052 | | | | | | 0.0142 | | | | | | * | |  |
| GP Role Other | | | | -0.3133 | | | | | | 0.1434 | | | | | | 0.0296 | | | | | | * | |  |
| Los 10+ | | | | 0.4708 | | | | | | 0.1876 | | | | | | 0.0125 | | | | | | * | |  |
| Residual standard error: 0.9483 on 367 degrees of freedom; Multiple R-squared: 0.05331, Adjusted R-squared: 0.04558; F-statistic: 6.889 on 3 and 367 DF, p-value: 0.0001592 | | | | | | | | | | | | | | | | | | | | | | | |  |
| Single term deletions: | | | | | | | | | | | | | | | | | | | | | | | |  |
|  | Df | Sum of Sq | | | | | RSS | | | | | | AIC | | | | Pr(>Chi) | | | | | | |  |
| <none> |  |  | | | | | 330.02 | | | | | | -35.420 | | | |  | | | | | | |  |
| Gender | 1 | 5.4568 | | | | | 335.48 | | | | | | -31.336 | | | | 0.01364 | | | | | | * |  |
| GP Role | 1 | 4.2889 | | | | | 334.31 | | | | | | -32.630 | | | | 0.02862 | | | | | | * |  |
| Length of service | 1 | 5.6634 | | | | | 335.69 | | | | | | -31.108 | | | | 0.01199 | | | | | | * |  |
| Significance codes: 0 ‘***’ 0.001 ‘**’ 0.01 ‘*’ 0.05 ‘.’ 0.1 ‘ ’ 1 | | | | | | | | | | | | | | | | | | | | | | | |  |
